# Multiple cellular effects of leaf extracts from *Parinari curatellifolia*

**DOI:** 10.1186/s12906-016-1287-6

**Published:** 2016-08-22

**Authors:** Mitchelle Gororo, Theresa Chimponda, Elaine Chirisa, Stanley Mukanganyama

**Affiliations:** 1 School of Pharmacy, College of Health Sciences, University of Zimbabwe, Mt. Pleasant, Harare, Zimbabwe; 2 Department of Biochemistry, University of Zimbabwe, Mt. Pleasant, Harare, Zimbabwe; 3 Bio-molecular Interactions Analyses Group, Department of Biochemistry, University of Zimbabwe, P.O. Box MP 167, Mt. Pleasant, Harare, Zimbabwe

**Keywords:** *Parinari curatellifolia*, Gout, Xanthine oxidase, Nitric oxide production, Apoptosis, Jurkat T cells, RAW 264.7 murine macrophages

## Abstract

**Background:**

*Parinari curatellifolia* is a prominent plant in folk medicine in Sub-Saharan Africa. The plant decoctions are used to treat various ailments, including the treatment of cancer, pneumonia, fever, microbial infections and anti-inflammation. The aims of the study were to investigate the effects of *P. curatellifolia* leaf extracts on cell inflammatory and proliferative activity.

**Methods:**

*Parinari curatellifolia* fresh leaves were collected from Centenary in Mashonaland Central Province of Zimbabwe. Plant extracts were prepared using methanol, water, acetone and ethanol. Firstly, the effects of the extracts were determined on xanthine oxidase activity. Kinetic constants were determined for the extracts that showed inhibitory effects. Then the effects of *Parinari curatellifolia* water extract on LPS, menadione and hydrogen peroxide-activated nitric oxide production in RAW 264.7 cells was determined by quantifying the amount of nitrites formed. Finally, the effects of *P. curatellifolia* on the proliferation of Jurkat-T cells as well as its modulation of cisplatin-induced cell- cytotoxicity was investigated on a Jurkat human T-cell lymphoma cell line.

**Results:**

There was significant XO inhibitory activity by the ethanol and methanol extracts at 15.6 μg/ml and 3.9 μg/ml respectively. The IC_50_ determination for allopurinol, ethanol extract and methanol extract were 0.43 μg/ml, 1.38 μg/ml and 2.19 μg/ml respectively. The kinetic results showed that the ethanol and methanol extracts were allosteric inhibitors of XO. The water extract of *P. curatellifolia* inhibited NO production in RAW cells when LPS was used as an activator. *P. curatellifolia* and cisplatin showed dose-dependent cytotoxicity on Jurkat-T cells. Isolated DNA from the cells showed that there was DNA cleavage on cells exposed to *P. curatellifolia* indicating that apoptosis may be a mechanism by which *P. curatellifolia* exerts its cytotoxicity on Jurkat-T cells*.*

**Conclusions:**

These results scientifically support the use of *P. curatellifolia* leaf extracts in the management of pain, inflammatory and neoplastic conditions. *P. curatellifolia* thus has multiple biological effects, thus, validating its use in traditional medical uses.

## Background

The use of medicinal plants and herbs is as old as the human species [1]. Mankind used plants and herbs to sustain their health [2]. There is a renewed interest in the use of natural therapeutic methods for the management of various conditions [3]. The use of herbal remedies is especially popular among the rural populace in developing countries and studies are being conducted to determine and improve medicinal principals in plants and herbs for use in the development of new pharmacotherapeutic agents [4]. In Zimbabwe, it is estimated that out of the 5000 species, about 10 % of them are being used as medicinal plants [5].

*Parinari curatellifolia* (Planth.ex Benth) belongs to the *Chrysobalanaceae* family under the genus name *Parinari. Parinari* is a vernacular name for a Brazilian species which means leaves like *Curatella* a West Indian and South American genus belonging to the *Dilleniaceae* family [6]. The *Chrysobalanaceae* consists of other genera such as *Licania, Exellodendron, Maranthes, Couepia, Hirtella, Acioa* and *Neocarya*. The genus *Parinari* consists of 32 species, with species that include *Parinari brasiliensis (Schott) Hook.f., Parinari capensis Harv.* and *Parinari occidentalis Prance. Parinari curatellifolia* is a valuable medicinal plant and different parts of the plant are used by traditional herbalists in the treatment of many medical conditions [1]. Amongst the uses of *P. curatellifolia* in ethnomedicinal practices is the use in dental hygiene where sticks are chewed [7], constipation, where the roots are crushed, mixed with hot water and liquid drunk [8]., hypertension, diabetes, liver related illnesses [9]. Eze and Wurochekke, [4] evaluated the curative effect of *P. curatellifolia* leaf extracts on inflammation of the epiglottitis. The leaf extracts showed antibacterial activity against strains such as Pseudomonas species and *Staphylococcus aureus* which are responsible for causing epiglottitis. The ethanol extracts of the root bark and leaves of *P. curatellifolia* were shown to be antibacterial against a number of strains such as *Escherichia coli, Staphylococcus aureus, Pseudomonas aeruginosa* and *Bacillus subtilis* [10].

Xanthine oxidoreductase (XOR, E.C. 1.17.3.2.) exists in two interconvertible forms namely xanthine oxidase (XO) and xanthine dehydrogenase (XDH) [11]. Xanthine oxidase plays a vital role in the metabolism of purine bases and related compounds from ingested nucleic acids to form uric acid. The enzyme oxidizes hypoxanthine to xanthine and then xanthine to uric acid. Other sources of purines are the salvage pathway resulting in cell death and the *de novo* synthesis of purines in the body [11]. The oxidized uric acid is then absorbed and excreted in urine [12]. Uric acid is insoluble in acidic environment, while its sodium urate salt is soluble at a pH near neutrality [13]. Hyperuricemia may be a result of overproduction or insufficient excretion of urates. Patients with hypouricemia exhibit xanthinuria or xanthine lithiasis. Lesch-Nyhan syndrome and Von Gierke’s Disease are associated with hyperuricemia [13]. Xanthine oxidase is also an important source of oxygen-derived free radical, these cause oxidative damage to living tissues, hence, initiating pathological processes such as inflammation, atherosclerosis, diabetic complications, cancers and aging [14]. Gout is one of the common metabolic disorders involving purine metabolism as a result of overproduction of uric acid [13]. The management of gout using conventional medicines entails the consumption of therapeutic agents such as xanthine oxidase inhibitors [15]. They block the biosynthesis of uric acid and they are purine analogues. Allopurinol is the preferred xanthine oxidase inhibitor clinically used. It was approved by the Food and Drug Administration in 1996 and it is used for prophylaxis of gout [11]. However, the use of allopurinol is associated with several side effects some of which are severe and may result in discontinuation of treatment. Some of these adverse effects are hypersensitivity reactions such as Steven Johnson syndrome, toxic epidermal necrosis, hepatotoxicity, neuropathy, haemolytic anaemia, visual disturbances among others [16]. Thus, there is interest for the development of new xanthine oxidase inhibitors with fewer side effects and better efficacy [11]. Alternative treatment of gout using ethnomedicines is becoming a common practice [17–19]. However, there is no scientific validation and there is inadequate documentation on the use of ethnomedicines from Zimbabwe as regards to pain and inflammation amongst other ailments [8].

The inhibition of inducible nitric oxide synthase is a possible target for regulation of inflammation. Inducible nitric oxide synthase (iNOS) produces larger proportions of NO in a shorter period of time compared to the constitutive forms of the enzyme. NO radical has a dual role in the immune response, first as an inflammatory effector molecule that activates other pathways and then as a free radical species that can be toxic to pathogens [20]. Both roles allow NO to be important in terms of chronic inflammation. A novel anti-inflammatory agent that would target iNOS specifically might significantly reduce occurrence of chronic inflammation and oxidative stress. The ideal effector agent for iNOS activity would alter its expression after activation of the immune system to discontinue the downstream effects of the NO radical [20, 21]. RAW 264.7 cell line is a line that was developed by transforming murine macrophages derived from a male mouse, with the Abelson murine leukemic virus [22]. The macrophage cell line is used as a model system to screen for anti-inflammatory agents. Macrophages play a central role in the initiation and propagation of the inflammatory process and serve as an interface between innate and adaptive immunity. The cells can be externally activated by antigens such as the *Escherichia coli* endotoxin, lipopolysaccharide (LPS) to produce inflammatory mediators and cytokines. Plants produce numerous antioxidant phytochemicals that protect plants against damage caused by active O_2_ formed due to exposure to ultraviolet radiation [23]. In this regard the role of the *P. curatellifolia* extract was studied to determine its effect on NO production in activated RAW 264.7 cells.

Chronic inflammation predisposes to different forms of cancer [24]. Use of non-steroidal anti-inflammatory agents is associated with protection against various tumours. Several chemopreventative agents are used to suppress the carcinogenic process [25] but they may cause toxicity and, thus, prevent their usage. There is a high death rate associated with cancer and because of the serious side effects of chemotherapy and radiation therapy, many cancer patients seek alternative methods of treatment [26]. Chemotherapy is one of the major treatment choices used for the control of advanced malignancies exhibits severe toxicity of normal tissues. With more than 1 500 anticancer drugs that are in active development, there is an urgent need to develop much effective and less toxic drugs [26]. The efforts of discovering anticancer agents from plants have had more success than other drugs [27]. There is a necessity for search of new compounds with cytotoxic activity as the treatment of cancer with the available anticancer drugs is often unsatisfactory due to the problem cytotoxicity to the normal cells [28]. The anticancer activity of *Parinari curatellifolia* was also investigated in this study since there is a link between inflammation and cancer [29]. The objectives of this study were to investigate the effects of *Parinari curatellifolia* leaf extracts on xanthine oxidase, nitric oxide production in RAW cells and anti-proliferative activity in Jurkat T cells in order to evaluate its claimed anti-inflammatory and anticancer effects.

## Methods

### Materials

#### Chemicals

All Chemicals for the following assays were purchased from were purchased from Sigma-Aldrich Chemicals, (Steinheim, Germany).

### Xanthine oxidase assay

The chemicals that were used in this assay were allopurinol, hypoxanthine, xanthine oxidase from bovine milk and the reagents namely potassium dihydrogen phosphate, potassium hydroxide, ethylene diamine tetraacetic acid disodium salt (EDTANa_2_), dimethyl sulfoxide (DMSO), nitroblue tetrazolium (NBT), sodium hydroxide and hydrochloric acid.

### Nitric oxide assay

The following were used in the nitric oxide assay: RAW 264.7 cell line, trypsin, Trypan Blue dye, menadione, hydrogen peroxide, lipolysaccharide (LPS), Dulbecco’s modified eagle medium (DMEM) supplemented with foetal bovine serum, antibiotics, indomethacin, iodine, potassium iodide, menadione, carbon tetrachloride, hydrogen, and Griess reagent (1 % sulphanilamide and 0.1 % N, (1-N-naphyl) ethylenediamine in 2.5 % phosphoric acid).

### Antiproliferative assay

The following reagents were used in the antiproliferative assay: Jurkat cells E 6.1 human leukemic T cell lymphocytes (ECACC, Sigma-Aldrich, Dorset, England), Rosewell Park Memorial Institute (RPMI) 1640 media, foetal bovine serum, antibiotics, penicillin, neomycin, streptomycin solution, Hanks Buffered Saline Solution, 1-chloro-2, 4-dinitrobenzene (CDNB), reduced glutathione (GSH), and cisplatin.

### Plant collection

*Parinari curatellifolia* fresh leaves were collected from Centenary (Latitute 16°48’00”S, Longitude 31°07’00”E and elevation above sea level is 1156 m) in Mashonaland Central Province of Zimbabwe in the summer period of February 2012. The plant samples’ identity were authenticated by Mr Christopher Chapano, a taxonomist at the National Herbarium located at the Harare Botanical Gardens, Harare, Zimbabwe. Voucher specimen (C6E7) were made and stored in the Biomolecular Interactions Analyses Laboratory at the Department of Biochemistry, University of Zimbabwe, and Harare, Zimbabwe. The leaves were washed with distilled water and then dried in an oven at 40 ˚C. The leaf powder was prepared using a blender (Philips Co., Shanghai, China). The leaf powder was stored in closed containers at room temperature.

### Preparation of plant extracts

The dried *P. curatellifolia* leaf powder was weighed using the KERN_EG_ balance (Kern and Son GmbH, D -72336 Balingen, Germany) and the following masses of 10 g, 10 g, 15 g and 15 g were mixed with the solvents methanol, water, acetone and ethanol respectively. A volume of 150 mls of each solvent was added to the leaf powder in a 1000 ml beaker. The beakers were placed on a magnetic stirrer and left stirring for 2 h. The solutions were filtered using Whatman No 1 filter paper (Sigma-Aldrich Chemicals, Steinheim, Germany) or transferred into syringe and filtered into a small glass vial. The sterile suspension was filtered again using 0.45 μM Millipore® sterile filter (Sigma-Aldrich, Taufkirchen, Germany) into a labelled small glass vial. The filtrate was evaporated to dryness, collected and stored at 4 ^o^C until when required.

### Xanthine oxidase assay

Xanthine oxidase activity assay used was adapted from Bergmeyer et al. [30] and with slight modifications. Initial screening for the effects of the water, methanol, acetone and ethanol extracts was done on the enzyme and the amount of uric acid produced was quantified spectrophotometrically at 295 nm using a UNICO^R^, UV/VIS 2800 spectrophotometer (United Products and Instruments Inc., U.S.A). The absorbance values obtained were proportional to the production of uric acid. A unit of activity for xanthine oxidase is that forming one micromole of urate per minute at 25 °C. The activity in the negative control that indicated 100 % enzyme activity was also determined for reactions without the extract. Appropriate volumes and concentrations of reactants: potassium phosphate buffer 50 mM pH 7.4, xanthine oxidase 0.72 U/ml, varying concentrations of the extract, and hypoxanthine 0.15 mM were added into a 1.5 ml quartz cuvette with an appropriate negative control. For the initial screening for inhibitory activity, five extract concentrations of 3.9, 15.6, 63,125 and 250 μg/ml were tested for their effects and the negative control contained no extract. For each of the concentration, the effect on activity was determined in quadruplicate. Superoxide anions are generated by both the hypoxanthine-xanthine oxidase and the xanthine-xanthine oxidase reactions. The superoxide anion radicals produced are detected by coupling them to reduction of nitroblue tetrazolium (NBT). A deep blue colour is produced when the superoxide react with NBT. Thus, colour intensity decreased with a decrease in xanthine oxidase activity. The IC_50_ determination method was adapted from Wang et al., [31]. Reagents were prepared in 50 mM potassium phosphate buffer of pH 7.4. The reagent mixture contained 20 μL of 15 mM EDTANa_2_ with pH 7.4, 50 μL of 0.6 mM NBT, 30 μL of 0.5 mM hypoxanthine, 50 μL of xanthine oxidase solution (0.06 U/ml), and 150 μL of various concentrations of plant extract or 150 μL of potassium phosphate buffer (as the control). For the kinetic reactions, a volume of 150 μL of three selected concentrations (0, 0.25, 0.5, and 1.0) μg/ml of allopurinol were added to the wells of the 96 microwell plate. For the extracts concentrations of 0, 1, 2, and 3 μg/ml were used. A volume of 150 μL of potassium phosphate buffer was then added followed by 20 μL of 15 mM EDTANa2, 30 μL of twelve different concentrations (0.01, 0.02, 0.03, 0.04, 0.05, 0.075, 0.1, 0.125, 0.150, 0.175, 0.2, 0.25) μg/ml of 0.5 mM hypoxanthine, 50 μL of 0.6 m nitroblue tetrazolium (NBT). For the methanol extract, the range of the concentration for hypoxanthine was from 0.01 to 0.3 μg/ml. The final concentrations of the other reagents in each well was 0.01 U/ml of xanthine oxidase, 0.1 mM of NBT and 1 mM of EDTANa2. The reaction was initiated by initiated by adding 50 μL of xanthine oxidase solution (0.06 U/ml) at 25 ^o^C, and absorbance values were measured every 30 s for 10 min at 550 nm using the Spectra Max Microplate spectrophotometer (Molecular Devices, Sunnyvale, U.S.A).

### Determination of kinetic constants for XO

Determination of kinetic constants for XO was carried out by the method of Wang et al., [31] with some modifications. All the reagents were dissolved in the 50 mM potassium phosphate buffer pH 7.4. A volume of 150 μL of three selected concentrations (0, 0.25, 0.5, and 1.0) μg/ml of allopurinol were added to the wells of the 96 microwell plate. A volume of 150 μL of potassium phosphate buffer (as the control), was added followed by 20 μL of 15 mM EDTANa_2_, 30 μL of twelve different concentrations (0.01, 0.02, 0.03, 0.04, 0.05, 0.075, 0.1, 0.125, 0.150, 0.175, 0.2, 0.25) μg/ml of 0.5 mM hypoxanthine, and 50 μL of 0.6 m nitroblue tetrazolium (NBT). The reaction was initiated by adding 50 μL of xanthine oxidase solution (0.06 U/ml, Sigma-Aldrich). The 96-microwell plate was incubated at 25 ^o^C in a shaking incubator (Jitterbug, 130000, Boekel Industries, Philadelphia, USA), and absorbance measured at 550 nm, readings were taken every 30 s for a period of 10 min using the Spectra Max Microplate spectrophotometer (Sunnyvale, U.S.A). The final concentrations of reagents in each well was 0.01 U/ml of xanthine oxidase, 0.1 mM of NBT, 0.05 mM of hypoxanthine and 1 mM of EDTANa_2_.

### Growth of RAW 264.7

Macrophage cell lines such as the RAW 264.7 cell line can be activated by external triggers to produce nitric oxide *in vitro*. A 1 ml aliquot of RAW cells was taken from vial and added to 10 ml of DMEM media and incubated at 37 °C and 5 % CO_2_. The spent media was decanted and fresh media was added and the vial was incubated at 37 ˚^o^C and 5 % CO_2_ in Shell Lab^®^ CO_2_ incubator (Sheldon manufacturing Inc., Cornelius, USA). The confluent cells underwent trypsinisation in order to conduct the cell count with the Trypan blue^®^ exclusion assay. A 200 μL aliquot of the trypsinised cells was mixed with 100 μL of Trypan blue^®^. The number of cells was determined using a haemocytometer. Viable cells would exclude the dye while dead cells would take up the dye appearing blue.

### Determination of the effect of *Parinari curatellifolia* ethanol leaf extract on nitric oxide production in menadione and hydrogen peroxide-activated RAW 264.7 cells

The effects of *P. curatellifolia* on nitric oxide production in RAW 264.7 cells after exposure to redox-active compounds menadione and hydrogen peroxide were investigated. The total number of RAW cells was enumerated as above and diluted to obtain a final cell concentration of 2 × 10 ^5^ cells/ml in the assay well of a 12-well plate. A volume of 1.9 ml of DMEM was dispensed into a microplate well and 1 ml of the cell suspension was added and mixed by gentle flushing. A 100 μL aliquot of the test compound was added to a final concentration of a 100 μM and this was followed by 25 μg/ml of *P. curatellifolia* the ethanol leaf extract. The concentration of 25 μg/ml was chosen as an appropriate non-lethal dose after carrying out a dose-dependent (15.6–250 μg/ml) -study of the effects of the extract. The plate was incubated at 37 ^o^C and 5 % CO_2_ in a Shell Lab^®^ CO_2_ incubator (Sheldon manufacturing Inc., Cornelius, USA) for 24 h. The contents of each well was centrifuged at 1500 rpm in a PLC-02^®^ benchtop centrifuge (Gemmy Industrial Corp., Taipei, Taiwan) for 10 mins. The cell free supernatants were measured by the nitrite quantification assay. The effect of the ethanol extract on the inhibition of the production of nitric oxide was determined by quantifying the amount of nitrites in the samples.

### Determination of the effect of *Parinari curatellifolia* on nitric oxide production in lipopolysaccharide -activated RAW 264.7 cells

Cells such as macrophages can express iNOS, the enzyme which is responsible for the production of large amounts of NO when stimulated by antigens such as the *Escherichia coli* endotoxin, lipopolysaccharide (LPS) [18]. The total number of RAW cells counted were diluted to a final cell concentration of 2 × 10^5^ cells/ml in the assay well of a twelve well plate. A 1.87 ml volume of DMEM was placed in a well and a 1 ml aliquot of the cell suspension was added and mixed. LPS was added to a final concentration of 100 ng/ml in the appropriate wells. The plant water extract at 25 μg/ml was added to the appropriate wells alone or in combination with LPS. The control wells contained media and cells only. The reaction plate was incubated at 37 °C and 5 % CO_2_ in a Shell Lab^®^ CO_2_ incubator (Sheldon manufacturing Inc., Cornelius, USA) for 24 h or 48 h. The contents of the wells were centrifuged at 1500 rpm in a PLC-02^®^ benchtop centrifuge (Gemmy Industrial Corp., Taipei, Taiwan) for 10 mins. The cell free supernatants were measured for the amount of NO produced by each experiment using the nitrite quantification assay described below.

### Quantification of nitric oxide

NO is converted to nitrite ions in the presence of oxygen. The principle of the assay is that the nitrite ions react with sulphanilamide in acidic conditions to a diazonium salt that in turn reacts with naphylethyldiamine. The reaction produces a pink azo product that absorbs maximally at 540 nm. A standard curve was prepared by two-fold serially diluting 100 μM solution of sodium nitrite in a microplate to a final concentration range of 20 to 1.25 μM. The contents of each sample well were centrifuged at 1500 rpm for 10 min. The cell free supernatants of the test samples were added to wells of the microplate. An equal volume of Griess reagent (1 % sulphanilamide and 0.1 % N, (1-N-naphyl) ethylnediamine in 2.5 % phosphoric acid) was added to the standard wells and test solutions and the plate was incubated for 10 min in the dark at room temperature. The reaction and standard curve samples were read in a Spectramax Plus^®^ UV–Vis microplate spectrophotometer (Molecular Devices Inc., California, USA) at 540 nm.

### The effect of glutathione on *P. curatellifolia* and cisplatin cytotoxicity

*P. curatellifolia* is used traditionally in the treatment of pain and fever and due to the link between cancer and inflammation, the anti-cancer effects of this plant were also determined on Jurkat cells, a human leukemic cell line. Jurkat cells E 6.1 human leukemic T cell lymphocytes (ECACC, Sigma-Aldrich, Dorset, England) were maintained in RPMI 1640 media supplemented with 10 % heat inactivated foetal bovine serum, 1 % penicillin, neomycin, streptomycin. Cells were grown at 37 °C in 5 % CO_2_ in a Shel Lab incubator (Sheldon MFG.INC, USA)_._ Jurkat-T cells (1 × 10^5^cells/ml) were seeded in 12-well plates and incubated in a CO_2_ incubator for 72 h. The cells were pre-treated with cisplatin (0–2 μg/ml) and *P. curatellifolia* ethanol extract (0–100 μg/ml). A total amount of 20 μl of the extract was used for the extract as well as cisplatin and each concentration was added in duplicate. The cells were counted on a haemocytometer after every 24 h. The effect of glutathione on *P. curatellifolia* and cisplatin cytotoxicity was also investigated. Jurkat T cells (1 × 10^5^) were seeded in 12 well plates and incubated in a CO_2_ incubator for 72 h. The cells were pre-treated with 50 μg/ml *P. curatellifolia* and 0.5 μg/ml cisplatin. The cells were pre-treated with the extract and GSH combined and cisplatin and GSH combined. Cells were also incubated with glutamate an inhibitor of glutathione and extract combined and cisplatin and glutamate combined. A control which had cells in RPMI media was also set up. The experiment was carried out in quadruplicate.

Jurkat cells (1 × 10^5^ cells/ml) were pre-treated with cisplatin (0–2 μg/ml), cisplatin (0–2 μg/ml) and 50 μg/ml *P. curatellifolia* maintained constant and cisplatin (0–2 μg/ml) and 12 μg/ml ethacrynic acid maintained constant. The cells were incubated at 37 °C for 72 h and then counted on a haemocytometer. Jurkat cells were collected by centrifugation at 2000 rpm for 5 min in a Hettich Rotofix 32 Centrifuge, (Tuttingen, Germany) and the supernatant was discarded. The cells were washed with 5 ml phosphate buffered saline and centrifugation was carried out at 2000 rpm for 5 minutes. The cells were lysed with 100 μl of lysis buffer (10 mM Tris (pH 7.4), 5 mM EDTA, 0.2 % Triton X-100) and 10 μl of 1 mg/ml proteinase K. The cells were incubated overnight at 56 °C in a water bath. A total of 4 μl RNA-ase (100 μg/ml) was added and the cells were further incubated at 37 °C for 1 h. Sodium chloride (1.5 M) was added as 1/10 the volume of the total volume and the tube was inverted several times. The tubes were then centrifuged at 12 000 rpm and the supernatant was retained into clean tubes. A 2 x volume of ice cold isopropyl alcohol was added to the DNA solution and the tubes inverted once more. The tubes were placed in a refrigerator for 1 h at -80 °C. The tubes were then centrifuged at 12 000 rpm for 15 min and the supernatant was discarded. The pellets were then washed with 70 % ethanol to remove any remaining contaminants. The ethanol was allowed to evaporate to leave the DNA. The DNA was re-suspended in TE buffer (10 mM Tris–HCl (pH 7.4) and 0.5 mM EDTA). The DNA was subjected to electrophoresis on a 1.5 % agarose gel at 100 V for 1 h.

### Statistical analyses

Statistically significant differences between the mean of the controls and the tests were analysed using one way ANOVA with Dunnett’s multiple comparison post-test. Enzyme kinetics was analysed with nonlinear regression and allosteric sigmoidal using GraphPad Prism5 (Version 5.03 GraphPad Software Inc. San Diego, California U.S.A).

## Results

### Effect of *P. curatellifolia* extracts on xanthine oxidase activity

**Fig. 1:**
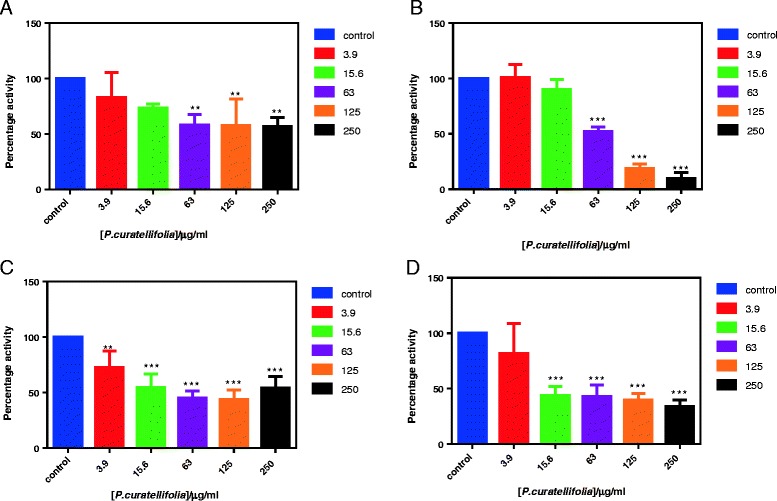
Effects of the *P. curatellifolia* water extract (**a**) acetone extract (**b**), methanol extract (**c**) and ethanol extract (**d**) on the activity of xanthine oxidase. Statistically significant differences (*p < 0.05, **p < 0.01 and ***p < 0.0001)

The acetone, ethanol, water and methanol extracts showed concentration-dependent inhibition of xanthine oxidase activity (Fig. 1). The water extract showed low xanthine oxidase inhibitory activity at low concentrations (3.9 and 15.6 μg/ml) but had a significant inhibitory activity at 63 μg/ml (Fig. 1a). Similarly, the acetone extract showed significant inhibition activity at a high concentration of 63 μg/ml (Fig. 1b) compared to the ethanol and methanol extracts. The ethanol and methanol extracts showed significant inhibitory activity at low extract concentrations. The methanol extract showed significant inhibitory activity at a concentration as low as 3.9 μg/ml, *p* value < 0.01 (Fig. 1c). While, the ethanol extract also showed significant inhibitory activity at 15.6 μg/ml, *p* value < 0.0001 (Fig. 1d). The IC_50_ values of the fractions that showed the statistically significant inhibitory activity namely the methanol and ethanol leaf extracts were determined. Extracts which showed statistically significant enzyme inhibition at concentrations less than 50 μg/ml qualify for further investigations [32]. Thus, methanol and ethanol extracts were selected for further investigations.

### IC_50_ Determination

**Fig. 2:**
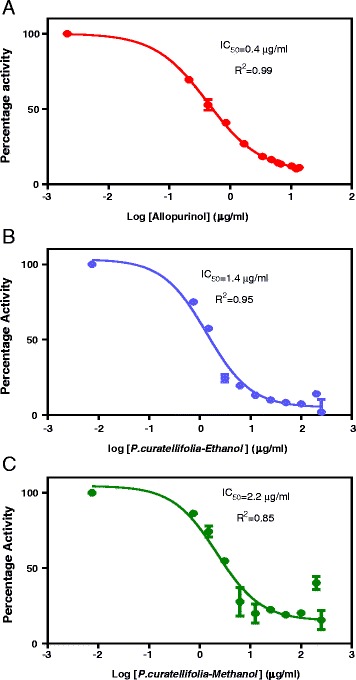
Determination of the IC_50_ values for the plants extracts and allopurinol. Graph **a** shows the results for allopurinol, graph **b** shows results for the ethanol extract and graph **c** shows results for the methanol extract

The IC_50_ of allopurinol was also determined as the positive control and it was compared with the IC_50_ values of the ethanol and methanol extracts (Fig. 2). Allopurinol was the most potent xanthine oxidase inhibitor with the lowest IC_50_ value. The ethanol extract followed allopurinol with an IC_50_ value of 1.38 μg/ml. Thus, the ethanol extract was more potent than the methanol extract which showed the lowest IC_50_ value.

### Determination of inhibition kinetics

**Fig. 3:**
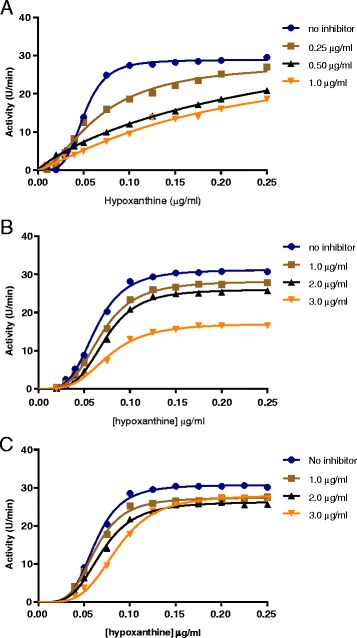
Kinetic determination of the methanol and ethanol extracts using allosteric sigmoidal. Graph **a**, **b** and **c** shows the effects of allopurinol, ethanol and methanol extracts respectively

The inhibition mechanisms of the ethanol and methanol *P. curatellifolia* leaf extracts were investigated where allopurinol was used as a positive control. The results obtained showed non-Michealis-Menten kinetics and so allosteric sigmoidal kinetics was used to analyse the results. The data was fitted onto the Hill equation and used to interpret allosteric enzyme kinetics. Figure 3 shows the shows a typical sigmoid patterns of the activity of the enzymes in the presence and absence of the extracts. The sum of the effects of the active compounds in the ethanol and methanol *P. curatellifolia* leaf extracts showed allosteric inhibition (Fig. 3b and c). On addition of the extracts a shift to the right was observed. This shift of the reaction kinetic graph to the right represents negative cooperativity meaning that as the extract concentration was increased, the affinity of the enzyme for its substrate hypoxanthine was decreased.

### Effects of *P. curatellifolia* extracts on activated RAW 264.7 murine macrophage cells

**Fig. 4:**
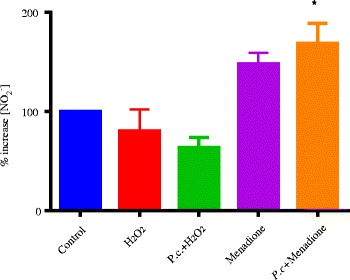
The effects of combining H_2_O_2_ or menadione and 25 μg/ml of *P. curatellifolia* water leaf extract on NO production. * denotes level of significance compared to the control of cells alone (*p* ≤ 0.05). The concentrations of H_2_O_2_ and menadione were 100 μM in each case

**Fig. 5:**
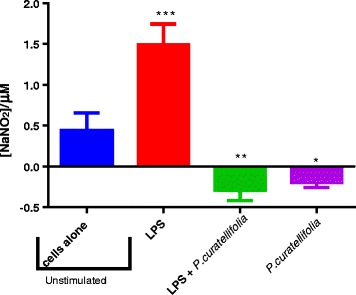
The effects of combining LPS and 25 μg/ml of *P. curatellifolia* water leaf extract on NO production. Nitrite concentration of RAW cells for the simultaneous combination of LPS with *Parinari curatellifolia* extract after 24 h. incubation are shown. * (*p* ≤ 0.05) versus control

RAW 264.7 macrophage murine cells were exposed to redox active compounds, menadione and hydrogen peroxide and a water extract of *P. curatellifolia* were added. Results show that menadione increased the levels of nitrite ions and this effect was further enhanced by incubation with 25 μg/ml of *P. curatellifolia* ethanol leaf extract (Fig. 4). Hydrogen peroxide had no effect on NO_2_ levels. Cells were activated for inflammation with the *E. coli* endotoxin, lipopolysaccharide (LPS), and exposed to 25 μg/ml of the water extract of *P. curatellifolia.* The addition of LPS to cells significantly increased the nitrite concentration from the baseline concentration of the un-stimulated RAW cells. The combination of LPS and *P. curatellifolia* water extract resulted in a significant decrease in the nitrite concentration when compared to both the un-stimulated cells and the LPS activated cells. *P. curatellifolia* water extract in control cells significantly decreased the baseline nitrite concentration (Fig. 5).

### Effect of *P. curatellifolia* on Jurkat cells

**Fig. 6:**
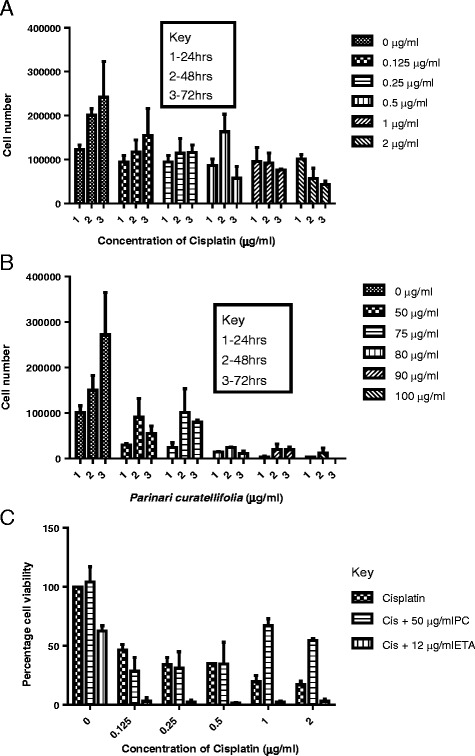
The effect of cisplatin (**a**) and *P. curatellifolia* (**b**) and (**c**) a combination of *P. curatellifolia* cisplatin and cisplatin and ethacrynic acid (ETA) on Jurkat T cells. There was a decrease in cell numbers with increase in concentration of the plant extract. The Figs. 1, 2 and 3 on the bar graphs represent the periods 24, 48 and 72 h

**Fig. 7:**
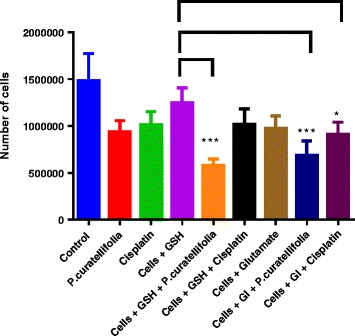
The effect of glutathione on the cytotoxicity of *P. curatellifolia.* The results are an average of four replicate values. The control contained the cells only which were incubated in RPMI media. *** *P* < 0.001. * *P* < 0.05

**Fig. 8:**
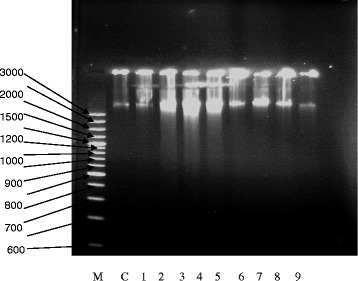
DNA ladder formation after exposure to cisplatin extract. From left Lane C: negative control, Lane 2–5 treated cells with different concentrations (50, 75, 90, 100 μg/ml) of P. curatellifolia ethanol extract after 72 h, Lane 6–9 treated cells with different concentration (0.125, 0.5, 1, 2 μg/ml) of cisplatin after 72 h, and M is a DNA Ladder (100 base pairs)

Cells were incubated with varying concentrations of cisplatin (0–2 μg/ml) and *P. curatellifolia* (0–100 μg/ml). The live cells after 24, 48 and 72 h were counted on a haemocytometer using trypan blue as a dye. Results are shown in Fig. 6. There was a decrease in cell density with increase in concentration of cisplatin and the plant extract (Fig. 6a and b). In addition, the combination of cisplatin and *P. curatellifolia* at higher concentrations if 1 and 2 μg/ml of cisplatin resulted in antagonistic effects in which cell viability actually increased (Fig. 6c). The effect of glutathione on the cytotoxicity of *P. curatellifolia* and cisplatin was investigated. The results show that incubation of cells with glutathione increased the cytotoxicity of *P. curatellifolia* extract and not of cisplatin. When incubated with glutamate, an inhibitor of glutathione synthesis, it was found that this amino acid enhanced that cytotoxicity of both the *P. curatellifolia* extract and cisplatin when compared to cells incubated with GSH and either of the compounds (Fig. 7). Thus, reduced glutathione augmented the anti-proliferative effects of *P. curatellifolia*. DNA was isolated from Jurkat-T cells and then subjected to electrophoresis on agarose gels. DNA fragmentation can be analysed by DNA ladder formation of isolated DNA in an electrical field. *P. curatellifolia* ethanol extract resulted in degradation of chromosomal DNA into nucleosomal fragments that is the biochemical hallmark of apoptosis (Fig. 8).

## Discussion

Plants are potential sources of lead compounds in search of new therapeutic agents for various clinical conditions [3]. *P. curatellifolia* has been seen to possess anti-bacterial, antifungal and anti-inflammatory effects [2]. The present study focused on validating the use of the plant in the treatment of gout, inflammation as well as determining its anticancer potential as inflammation plays a significant role in carcinogenesis [33]. The ethanol, water, acetone and methanol leaf extracts of *P. curatellifolia* were prepared and the effects of these extracts on the activity of xanthine oxidase, raw macrophage and Jurkat T cells were determined.

Xanthine oxidase is a versatile and complex molybdenum-and iron-containing flavoprotein [34] that catabolizes the rate limiting step in the catabolism pathway of purines [35]. A two-step reaction is involved in which hypoxanthine is oxidized to xanthine then xanthine oxidized to uric acid. At physiological pH uric acid loses a proton and forms urate, the urate is then excreted in urine [34]. Allopurinol is a purine analogue that is used as prophylaxis in the management of hyperuricemia and gout. Allopurinol acts by competitively inhibiting the activity of xanthine oxidase and purine biosynthesis. This results in the reduction of uric acid production [13]. Allopurinol is the commonly used xanthine oxidase-inhibitor that is available commercially. However; it is associated with various side effects some of which are fatal such as Steven Johnsons’ Syndrome, thrombocytopenia, toxic epidermal necrolysis syndrome, liver function abnormalities, leukocytosis, eosinophilia, vasculitis, aseptic meningitis, renal dysfunction and hepatic dysfunction [36].

The xanthine oxidase inhibitory activity assay was conducted and the results showed that all of the four extracts (ethanol, methanol, water and acetone) inhibit xanthine oxidase in a concentration-dependent manner. However, the ethanol and methanol extracts showed significant inhibitory effects at concentrations of 15.6 μg/ml and 3.9 μg/ml respectively. The IC_50_ determination assay results showed the order of potency to be allopurinol > ethanol extract > methanol extract. The results of xanthine oxidase kinetics showed that inhibition by the extracts followed non-Michaelis-Menten kinetics with allosteric model being the best fit mode.

There was positive cooperativity in the activity of xanthine oxidase using hypoxanthine as the substrate. On addition of the extracts, the curves shifted to the right with increase in the extract concentration. A shift to right represents negative cooperativity which means that the affinity of xanthine oxidase for hypoxanthine decreased with an increase in the extract concentration. A decrease in affinity for hypoxanthine results in a decrease in uric acid production (Fig. 3). Several studies are being conducted with the effort of trying to find a new xanthine oxidase inhibitor which are more efficacious than allopurinol and with fewer side effects. A new bisflavonoid (loniceraflavone), which is the active principle of *Lonicera hypoglauca* was isolated. This compound was found to be a potent inhibitor of xanthine oxidase [37]. This is in agreement with other studies that have shown that xanthine oxidase inhibitory activity was contributed by flavonoid compounds [38]. Azmi et al. [17], also stated that polyphenols, flavonoids, coumarins, ellagic acid and valoneic acid dilactone have been reported to be potent xanthine oxidase inhibitors. Lignans, iridoids, a chalcone compound have also been reported to possess xanthine oxidase inhibitory activity [38]. The presence of phenolics and flavonoids in the extracts of *C. adansonii* contributed towards the inhibitory activities observed [39]. This study also showed that *P. curatellifolia* leaf extracts inhibited xanthine oxidase activity in a concentration-dependent manner. When *P. curatellifolia* leaf extracts are used traditionally in management of pain and inflammation, the mechanism of action may partly be due to its inhibitory effect on xanthine oxidase.

**Fig. 9:**
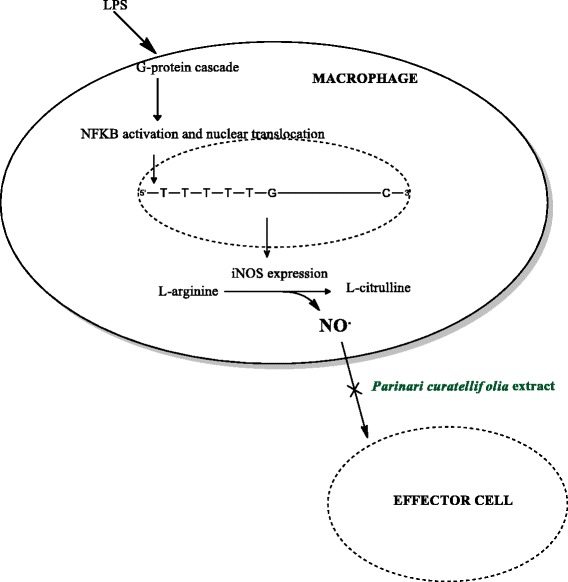
Proposed mechanism of action of effect of *Parinari curatellifolia* water extract on nitric oxide production in RAW 264.7 murine macrophage cells. Lipopolysaccharide (LPS) activates a G-protein cascade that leads to the dissociation of NFKβ from its inhibitor molecule IKβ. The dissociation of NFKβ allows nuclear translocation of the transcription factor to activate the expression of iNOS. iNOS catalyses the breakdown of L-arginine to citrulline with NO as a by-product. *P. curatellifolia* water extract was able to scavenge NO radicals which could reduce its effect on its target cells

Oxidative stress plays an important role in the development of chronic inflammatory conditions. Macrophages play important signalling and therapeutic roles when they produce and release nitric oxide during an inflammatory event. The nitric oxide is eventually converted to the more stable nitrite form. Due to the short half-life of NO, the concentrations of the radical produced are usually measured by quantifying the amount of nitrites present in the samples. NO production in the RAW cells was activated using an *E. coli* endotoxin, lipopolysaccharide [18]. LPS has been shown to induce the expression of iNOS in macrophage cells in cell culture leading to an increase in the production of NO within the cells. The effects of *P. curatellifolia* water extract on the NO production was investigated in this study. *P. curatellifolia* water extract was incubated with the RAW cells in the presence and absence of the endotoxin LPS. The fact that *P. curatellifolia* water extract has on nitric oxide production in RAW cells implies that the extract has inflammatory activity by quenching the NO radicals. The proposed mechanism shown in Fig. 9 proposes that the NO radicals are removed by the water extract before they are converted to the nitrite form. The proposal for the mechanism was based on two pieces of evidence. The first piece of evidence is that there was a decrease in the nitrite concentration in the un-induced RAW cells in the presence of *P. curatellifolia* water extract indicating a reduction in the baseline NO concentration. The LPS induced cells showed a significant increase in NO production compared to the un-induced cells. The combination of LPS and *P. curatellifolia* water extract led to a significant decrease in the nitrite concentration compared to the LPS activated cells. The second piece of evidence is that *P. curatellifolia* water extract was found to be a potent scavenger of the NO radical in a study by Boora et al., [40]. *P. curatellifolia* extracts of seeds and pulp were found to possess antioxidant activity against the DPPH radical [41, 42]. However, the extract caused a decrease in the baseline nitrite concentration which is required for normal physiological roles such as neurotransmission, vascular flow and synaptic plasticity [43]. The inhibition of NO production required for the physiological functions could result in toxicity if administered in the case of an overdose. Therefore, the use of the *P. curatellifolia* extract as an herbal remedy might require proper formulation to ensure that either the therapeutic or chemo preventive concentrations are administered. The extract could be fractionated by bio-guided fractionation to determine and isolate the active components if there is no loss in potency.

The NO scavenging activity of the *P. curatellifolia* water extract may be responsible for the anti-inflammatory activity of the decoctions that are administered by herbalists in the treatment of stomach-aches and fever. The study investigated the effect of the water extract of *P. curatellifolia* on nitric oxide production, which is likely to be similar to the decoction that is administered by herbalists. Most herbal remedies are prepared in water. Therefore, the results support the use of *P. curatellifolia* as a pain remedy in folk medicine. The *P. curatellifolia* water extracts shows potential as a source of NO radical scavenging agents that could have anti-inflammatory activity. Thus, *P. curatellifolia* water extract could directly reduce the levels of NO in RAW cells and that could be its mechanism of action when used as an anti-inflammatory agent in folk medicine. Other studies have also shown that *P. curatellifolia* has inhibitory effects on HPGDH_2_ which is involved in inflammation [44].

*P. curatellifolia* reduced the growth of Jurkat T cells in a dose-dependent manner. The anti-proliferative effect of plant extracts from Zimbabwe against Jurkat cells and Wil 2 cell lines was also investigated previously [45]. *P. curatellifolia* methanol extract at a concentration of 10 μg/ml was shown to reduce cell proliferation of Jurkat T cells by 70 % after 48 h of incubation. The anti-proliferative effect of *P. curatellifolia* ethanol extract was also demonstrated and further experiments to determine its mechanism of action were carried out. Two structurally cytotoxic ent-kaurene diterpenoids, 13-methoxy-15-oxozoapatlin and 13-hydroxy-15-oxozoapatlin, were isolated from the root bark of *P. curatellifolia*, together with the known compound, 15-oxozoapatlin, and found to demonstrate broad-spectrum cytotoxic activity against a panel of cultured human cancer cell lines [46]. The cytotoxicity of the crude methanol extract of *P. curatellifolia* against a Hela cell line was attributed to its high polyphenolic content [2]. The polyphenolics may be responsible for the cytotoxicity of the ethanol extract of *P. curatellifolia* against Jurkat cells since as it is a polar solvent like methanol. Further studies were done to determine the possible mechanism of action of *P. curatellifolia* on cancer cell lines.

*P. curatellifolia* is a plant used in Nigerian folk medicine for treatment of cancer and other diseases [2]. In this study, the effect of reduced glutathione on Jurkat cells in the presence and absence of cisplatin was investigated in order to determine if the mechanism of action involved a redox reaction. *P. curatellifolia* and cisplatin cytotoxicity were not affected by the presence of glutathione. In a previous study, GSH did not change the inhibiting effects of oxaliplatin and cisplatin on lung carcinoma A549 cell line proliferation [47]. GSH depletion was shown to increase cell sensitivity to Fas-induced apoptosis [48]. L-*S*,*R*-buthionine sulfoximine (BSO) is a potent specific inhibitor of γ-glutamylcysteine synthetase, the rate-limiting enzyme in GSH biosynthesis, and has been used to deplete intracellular GSH and to reverse drug resistance in tumour cells [48]. Glutamate was used in order to deplete the GSH in the study. *P. curatellifolia* cytotoxicity improved but the cytotoxicty of cisplatin in Jurkat cells remained unchanged in the presence of glutamate. In this study, the effects of *P. curatellifolia* ethanol extract were antagonised by cisplatin whilst previously we have shown that the combination of doxorubicin with *P. curatellifolia* methanol extract was shown to increase the anti-proliferative action of doxorubicin [45]. This shows that if a plant extract is antagonistic with one drug, it may have synergy with another drug highlighting the importance of further studies of a plant extract in combination with other anticancer agents. In further exploration of the mechanism of action of P*. curatellifolia, in vitro,* the effects of DNA integrity were studied. Exposure of Jurkat T cells to *P. curatellifolia* extracts resulted in the fragmentation of DNA into small nucleosome ladders. It has been reported that at times it may be difficult demonstrate the presence of as they may only been clearly when large numbers of cells die in synchrony [49]. This is evidence for the induction of apoptosis, one of the mechanisms by which the extract may induce its cytotoxicity. The biochemical hallmark of apoptosis is the degradation of the genomic DNA, an irreversible event that commits the cell to die and occurs before changes in plasma membrane permeability [50].

## Conclusion

In conclusion, our study has shown that *P. curatellifolia* leaf extracts showed xanthine oxidase inhibitory activity and these results scientifically support the use of *P. curatellifolia* leaf extracts in the management of pain as per traditional medical practices. *P. curatellifolia* water extracts was also shown to be a potent inhibitor of nitric oxide production in RAW cells that could be its mechanism of action as an anti-inflammatory agent in folk medicine. Epidemiological research has clearly established that chronic inflammation is linked to approximately 15 %–20 % of cancers, including and especially colorectal cancer. *P. curatellifolia* may this serve as a source a potential source of compounds that may serve as anti-inflammatory agents and directly and indirectly as anticancer agents.
